# Investigating the accuracy of Garmin PPG sensors on differing skin types based on the Fitzpatrick scale: cross-sectional comparison study

**DOI:** 10.3389/fdgth.2025.1553565

**Published:** 2025-03-27

**Authors:** Annie Icenhower, Claire Murphy, Amber K. Brooks, Megan Irby, Kindia N’dah, Justin Robison, Jason Fanning

**Affiliations:** ^1^ Department of Health and Exercise Science, Wake Forest University, Winston-Salem, NC, United States; ^2^ Department of Anesthesiology, Wake Forest University School of Medicine, Winston-Salem, NC, United States

**Keywords:** physical activity, wearables, heart rate, equity, exercise

## Abstract

**Background:**

Commercial wearable devices, which are often capable of estimating heart rate via photoplethysmography (PPG), are increasingly used in health promotion. In recent years, researchers have investigated whether the accuracy of PPG-measured heart rate varies based on skin pigmentation, focusing particularly on the accuracy of such devices among users with darker skin tones. As such, manufacturers of wearable devices have implemented strategies to improve accuracy. Given the ever-changing nature of the wearable device industry and the important health implications of providing accurate heart rate estimates for all individuals no matter their skin color, studies exploring the impact of pigmentation on PPG accuracy must be regularly replicated.

**Objective:**

We aimed to contrast heart rate readings collected via PPG using the Garmin Forerunner 45 in comparison with an electrocardiogram (ECG) during various levels of physical activity across a diverse group of participants representing a range of skin tones.

**Methods:**

Heart rate data were collected from adult participants (18–64 years of age) at a single study session using the Garmin Forerunner 45 PPG-equipped smartwatch and the Polar H10 ECG chest strap. Skin tone was self-reported via the Fitzpatrick scale. Each participant completed two 10 min bouts of moderate-intensity walking or jogging separated by a 10 min bout of light walking.

**Results:**

A series of mixed ANOVAs indicated no significant interaction between Fitzpatrick score and phase of the activity bout (i.e., rest at the start, first intensity ramp-up phase, first steady-state phase, active rest, second ramp-up phase, and second steady-state phase). Similarly, there was no significant main effect for the Fitzpatrick score, although there was a significant main effect for phase, which was driven by greater ECG-recorded heart rate relative to PPG during the first ramp-up phase.

**Conclusion:**

Our findings support prior research demonstrating no significant impact of skin tone on PPG-measured heart rate, with significant differences between PPG- and ECG-measured heart rate emerging during dynamic changes in activity intensity. As commercial heart rate monitoring technology and software continue to evolve, it will be vital to replicate studies investigating the impact of skin tone due to the rapidity with which widely used wearable technologies advance.

## 1 Introduction

Regular physical activity promotes overall health and well-being across the lifespan and accompanies improvements in cognition, learning and judgment skills, and symptoms of depression and anxiety (1, 2). According to a 2022 report from the World Health Organization, more than 80% of the world's adolescent population is insufficiently physically active, and people who do not engage in sufficient physical activity are at 20%–30% greater risk of death compared to sufficiently active people (3). Wearable activity monitors have risen in popularity both for consumers and researchers given their ability to objectively monitor key health behaviors including physical activity, sleep, and stress, which are each facilitated based on heart rate monitoring (4, 5). These are useful technologies for those interested in activity promotion, as a core component of successful activity behavior change is self-regulation, which is characterized by the motivation, control, and modification of behavior to achieve a desired goal (6). Successfully changing behavior through self-regulation depends on one's ability to accurately and consistently self-monitor their behaviors, as developing an accurate awareness of one's behaviors is a pre-requisite for supporting behavior change.

Consumer physical activity self-monitoring technologies represent an important and growing industry (7–9) and are increasingly common in clinical physical activity trials (10–12). Contemporary monitoring devices integrate various sensing technologies such as accelerometry, global positioning, and optical sensing to measure the intensity and duration of activity. These data are of immense value to those interested in developing novel and highly tailored activity programs. However, concern over whether sensors work similarly across individuals—and the potential to introduce a systematic bias when using monitoring technologies—has risen in popular consciousness in recent years. Wrist-worn consumer devices leverage light (photoplethysmography; PPG) to monitor peripheral blood flow, and there is concern that darker skin tones may affect the accuracy of PPG sensors (13, 14). A systematic review by Koerber and colleagues indicated that heart rate-sensing smartwatches were significantly less accurate when used on darker skin tones in comparison to lighter skin tones (13). In contrast, Bent and colleagues failed to identify significant differences in accuracy resulting from differing skin tones, though they did find that accuracy varied by device manufacturer and type of activity, regardless of skin tone (14).

One potential cause of heterogeneity in findings in the relationship between skin tone and PPG accuracy is the continuous evolution of heart rate monitoring hardware and software in the consumer market. This presents both challenges (e.g., changing study endpoints) and benefits (e.g., enhanced accuracy) in the research context. For instance, companies such as Garmin and Apple have implemented technologies to increase the intensity of the PPG light if a strong signal is not detected by the device, such as for individuals with darker skin (15). Because consumer technologies are continuously refined and improved upon, replication studies are critical—a notion increasingly recognized in mHealth research (16). To this end, the purpose of this study was to revisit the investigation of differences in electrocardiogram (ECG) and optical heart rate (PPG) data collected by Garmin Forerunner 45 and Polar H10 devices, respectively, across self-reported skin tone scores.

## 2 Methods

Participants with varying skin tones were recruited between Spring 2022 and Spring 2023 to compare heart rate recordings during rest, exercise, and recovery between wrist-worn PPG and chest ECG. Participants were recruited via word of mouth, flyers, and listserv emails within a college community in the southeast United States. Participants were eligible if they were between 18 and 64 years of age with no forearm tattoos that would interfere with the PPG sensors on both wrists. Participants were intentionally recruited such that no more than half of the sample self-identified as White. In addition, eligible participants had to be fluent in English, capable of communicating with research staff over the phone, willing to wear two Garmin wristwatches and a chest strap for approximately 35 min, and able to engage in aerobic exercise for at least 20 min. Eligible participants completed a pre-screen interview including a physical activity readiness questionnaire to confirm eligibility and subsequently were scheduled for a single testing session. At the session, and prior to all study procedures, research staff conducted the informed consent process, obtained written consent from the participants, and collected participant demographic characteristics and the Fitzpatrick scale as a proxy for skin tone.

PPG data were collected via the Garmin Forerunner 45, which leverages the same Garmin Elevate PPG technology used across all modern Garmin devices (17). We selected this device as Garmin devices are well-represented in both physical activity research (18) and in the commercial sectors (19, 20). Additionally, because Garmin devices all leverage the same PPG technology, selecting a single device equipped with this sensor offers an efficient means of investing in PPG accuracy in a large segment of the consumer wearable market. We want to emphasize the importance of expanding the work presented in this study to other widely used consumer devices. Participants were fitted with one Garmin Forerunner 45 on their left wrist and another on their right wrist. One Garmin collected data via the PPG sensor, and the second was connected to a Polar H10 ECG chest strap and therefore did not collect data via PPG. Electrode gel or water was placed on the sensors of the chest strap before being fastened to the participant. Participants remained seated for 5 min to collect their resting heart rate. Resting heart rate was then used to calculate heart rate reserve (HRR), which was computed by subtracting resting heart rate from the participant's estimated maximum heart rate, which was calculated using the formula: estimated maximum heart rate (BPM) = 220-age (21). Participants were then instructed to walk or jog on an outdoor track at 60% of their estimated HRR for 10 min during an “exercise bout.” Participants then walked at a self-selected light intensity for 10 min (i.e., “rest”) and then initiated a second “exercise bout” wherein participants again were instructed to walk or jog at 60% of their estimated HRR for 10 min. We selected this protocol to give insight into both steady state and changing intensity given prior research demonstrating differential performance of PPG vs. ECG during intensity changes (14). Upon completion of the participant visit, data were downloaded from each Garmin Forerunner 45 and extracted via a custom Python script (22).

## 3 Measures

### 3.1 Heart rate

As noted above, Garmin Forerunner 45 devices were used to collect data during each session, with one paired to a Polar H10 ECG chest strap and the other using the on-device PPG sensor. The Polar H10 is among the most widely used chest ECG devices with excellent validity (23). Heart rate data are provided approximately every 5 s, and a custom Python script was utilized to time-match data collected via both devices. Specifically, datasets were merged based on closest matching timestamps, which were allowed to differ by up to 5 s. These data were subsequently plotted, and periods of rest, the first exercise bout, the rest bout, and the second exercise bout were identified with a timestamp and visual inspection of ECG data. As it has been reported that PPG data may be delayed relative to ECG data during changes in activity intensity (14), we investigated exercise bouts as a whole as well as subdivided into a “ramp” and “steady-state” period. Specifically, we visualized ECG-based heart rate data and classified the rapid increase in heart rate during the initial period of the exercise bout as the “ramp” period and the plateau in heart rate as “steady state.” If a participant did not have a clear delineation between these stages (e.g., a consistent rise in heart rate across the bout), all activity was classified as exercise. In sum, PPG and ECG differences in a total of eight “phases” were investigated: rest, the first ramp, the first steady-state exercise bout, the first full exercise bout (comprising both the ramp and steady-state period), rest, the second ramp, the second steady-state exercise bout, and the second full exercise bout (comprising both the ramp and steady-state period). Average readings and differences for the ECG and PPG data were computed for each period. Differences were computed as ECG minus PPG, such that positive values indicated higher ECG-measured heart rate whereas negative values indicated higher PPG-measured heart rate. Notably, the Association for the Advancement of Medical Instrumentation recommends a maximum error of ±5 BPM for heart rate monitoring (24).

### 3.2 Skin tone

The Fitzpatrick scale was originally designed to classify how different skin types may react to ultraviolet light, though as Fine and colleagues note, the Fitzpatrick scale “is often used within the biophotonics community due to the effect eumelanin has on how light travels through skin. This is due to the high absorbance of eumelanin with a peak in the ultraviolet wavelength (220 nm) and a steady decay through the visible wavelength region” (25). Participants responded to 10 items related to physical traits (e.g., eye color and color of the skin in unexposed areas), sensitivity to sun exposure, and how often one typically engages in intentional sun exposure. Responses are provided on a 0–4 scale, with final scores ranging from 0 to 40. Six total categories are derived from these scores, ranging from pale white skin to deeply pigmented dark brown skin to black skin (26). In the present study, we investigated Fitzpatrick scores continuously (14, 27) as well as in three categories containing two Fitzpatrick types each (i.e., 0 = types I/II, corresponding to scores of 0–13; 1 = types III/IV, corresponding to scores of 14–27; 2 = types V/VI, corresponding to scores of 28–36). This scale is commonly used in research and clinical settings to classify one's skin tone (28), largely due to its availability, historic use, and ease of administration. However, while it is frequently used by healthcare providers as a means of describing skin color (29), it is notable that it was originally designed to measure the propensity of the skin to burn during phototherapy (29). Important limitations to this approach include that Fitzpatrick is often conflated with a measure of race or ethnicity and that there is a large degree of within-group variability in skin tone (29). We deem it important to note early that the use of the Fitzpatrick scale is a limitation driven by a lack of widely available tools and will explore opportunities for future research within the discussion.

We leveraged a series of descriptive analyses to contrast heart rate collected via chest ECG and wrist PPG among individuals with varying skin tones assessed via the Fitzpatrick scale. First, we present descriptive statistics, including mean (SD) for continuous variables and count (%), for the whole sample. Similarly, we computed descriptive statistics for the difference in heart rate within each phase of the exercise bout (start, first ramp, first steady-state exercise, first full exercise bout, rest, second ramp, second steady-state exercise, second full exercise bout), with descriptives presented for Fitzpatrick subgroups and the sample as a whole. Note that we observed two extreme outliers in the difference between ECG and PPG-recorded heart rate, and as such, we also present median and interquartile range in supplemental materials. Both individuals fell into the Fitzpatrick type V/VI category. Bland–Altman plots were produced for each phase to investigate differences in ECG and PPG heart rate by average heart rate. To investigate whether heart rate varied by Fitzpatrick score, phase of the exercise bout, or the interaction of the two, we next conducted a mixed ANOVA including phase as a within-subject factor and Fitzpatrick subgroup as a between-subject factor, confirming the assumption of homogeneity of variances. Finally, to investigate relationships between continuous Fitzpatrick scores and differences in heart rate by device during each phase, we computed a series of Pearson correlations that were interpreted as recommended by Evens and colleagues such that 0–0.2 was considered very weak, 0.2–0.4 was considered weak, 0.4–0.6 was considered moderate, 0.6–0.8 was considered strong, and 0.8+ was considered very strong (30); significant associations were visualized via scatterplot. Given the outlying values described above, we also present Spearman rho correlations in the supplemental materials. All analyses were completed in SPSS version 29 (IBM Corp., Armonk, NY, USA).

## 4 Results

Participant characteristics are displayed in Table 1. A total of 33 participants agreed to participate in the study, and 29 completed the study protocol and had complete data from both devices during the 1-year pilot period. Characteristics of these participants are displayed in Table 1. Briefly, 8 identified as Black, 4 as Asian, 15 as White, and 2 self-identified as more than one race. With regard to sex, 15 participants (52%) were male, and 14 (48%) were female. The average age of the participants was 22.24 ± 4.54 years, and the average Fitzpatrick score from the research sample was 21.45 ± 6.48.

**Table 1:** Participant demographics (*N* = 29).

Characteristic	M (SD)
Age, M (SD)	22.24 (4.54)
Male, *n* (%)	15 (52)
Female, *n* (%)	14 (48)
Race, *n* (%)	
White	15 (52)
Black	8 (28)
Asian	4 (14)
More than 1	2 (7)
Fitzpatrick score, M (SD)	21.45 (6.48)
BMI M (SD)	23.19 (3.60)

M, mean; SD, standard deviation; BMI, body mass index (kg/m^2^).

**Figure 1:**
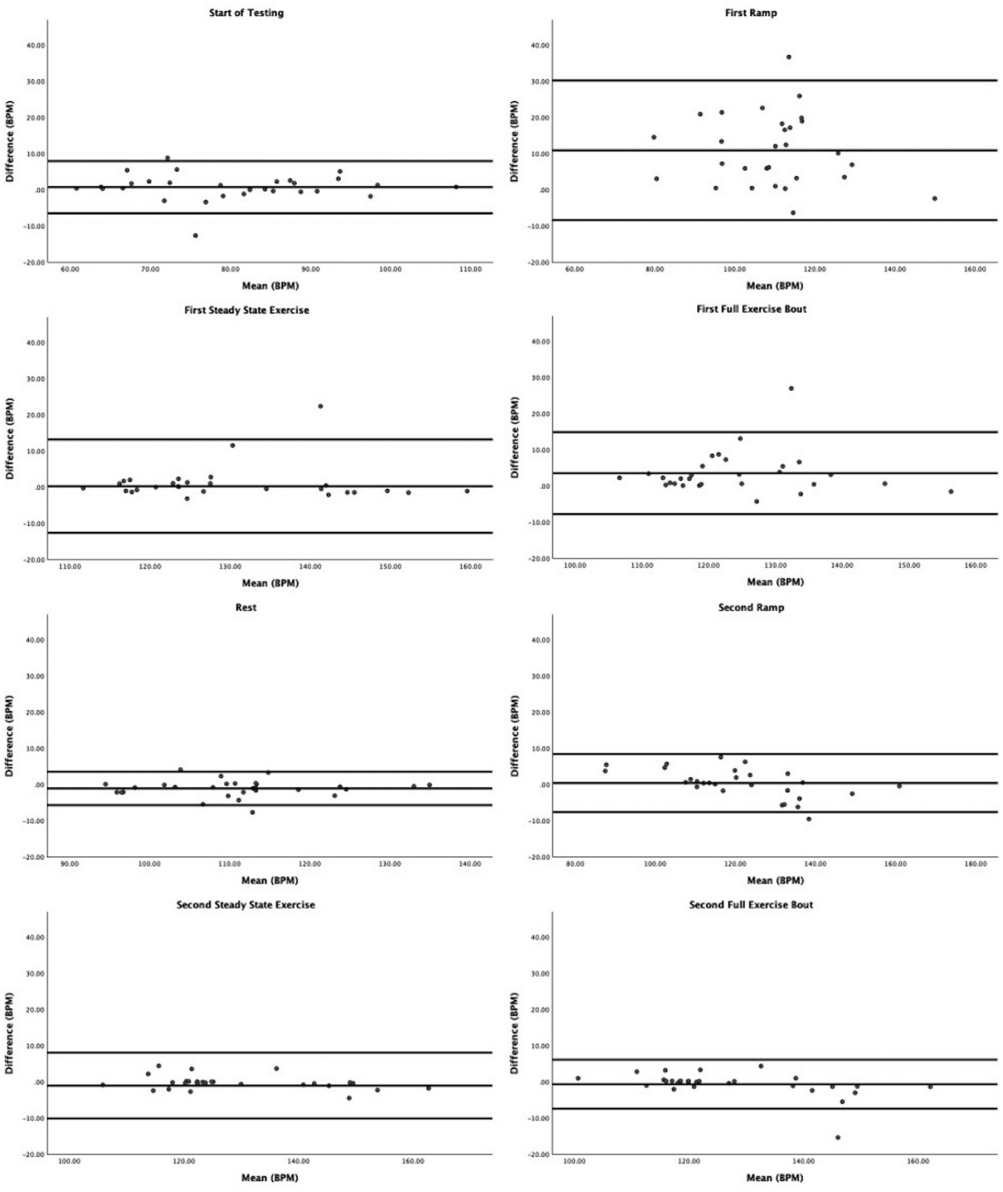
Bland–Altman plots contrasting differences in ECG vs. PPG by average across both devices. The lines are plotted for average difference and 95% confidence limits around the mean difference.

Table 2 depicts the descriptive statistics during each phase by Fitzpatrick category. Supplementary Table S1 contains median and interquartile range values during each phase by Fitzpatrick category. Figure 1 depicts the Bland–Altman plots for each phase. Note that there was a violation of the sphericity assumption for the mixed ANOVA, and therefore we corrected degrees of freedom using the Greenhouse-Geisser *ɛ* = 0.509. The mixed ANOVA did not reveal a significant phase–Fitzpatrick category interaction (*P* = 0.27), nor was there a significant main effect for the Fitzpatrick category (*P* = 0.68). There was, however, a significant main effect for phase [*F*_(2.55,63.64)_ = 19.84, *P* < .001, *η*^2^ = .44], and a series of *post hoc* contrasts revealed this was driven by significantly higher ECG-recorded heart rate relative to PPG during the first ramp phase, which was significantly larger than differences during any other phase (*P* < .001). Differences in ECG and PPG between other phases were not statistically significant. A second model wherein ramp and steady-state phases were not separated demonstrated similar results. Namely, the interaction between the Fitzpatrick category and phase was not significant (*P* = 0.21) nor was the main effect for the Fitzpatrick category (*P* = 0.57). The main effect for phase was significant [*F*_(2.16,56.13)_ = 8.36, *P* < .001, *η*^2^ = .24], and this was driven by significantly greater differences in the first exercise phase (*P*s ≤ 0.006). Notably, several individuals had at least one extreme outlying value during at least one phase. As sensitivity analyses, we conducted the mixed ANOVAs without the outlying values, and the interpretation did not differ.

**Table 2:** Mean and standard deviations for the difference of heart rate in each task overall and by Fitzpatrick category.

Phase	Fitzpatrick score			Total (*N* = 29)
	0–13 (*N* = 5)	14–27 (*N* = 17)	28–36 (*N* = 7)	
Start	−1.51 (6.49)	1.58 (3)	0.04 (1.97)	0.67 (3.67)
First ramp	14.35 (7.00)	8.48 (9.06)	13.99 (12.66)	10.82 (9.82)
First steady-state exercise	−0.08 (1.97)	−1 (6.48)	3.32 (8.43)	0.2 (6.57)
First full exercise bout	5.01 (3.59)	1.84 (3.87)	6.42 (9.32)	3.49 (5.76)
Rest	−1.00 (1.28)	−1.25 (2.78)	−1.01 (1.99)	−1.15 (2.35)
Second ramp	2.51 (3.77)	0.89 (3.45)^a^	−2.49 (4.62)	0.34 (4.07)^a^
Second steady-state exercise	−0.23 (2.33)	−1.56 (5.72)	−0.64 (2.69)	−1.11 (4.62)
Second full exercise bout	0.42 (2.35)	−0.92 (3.97)	−0.97 (2.89)	−0.7 (3.44)

Differences were computed as ECG − PPG such that more positive scores indicate higher ECG-recorded heart rate. Values are in average beats per minute.

^a^ One observation missing due to a lack of clear delineation between the ramp and steady-state exercise and therefore time was all categorized as exercise.

**Figure 2:**
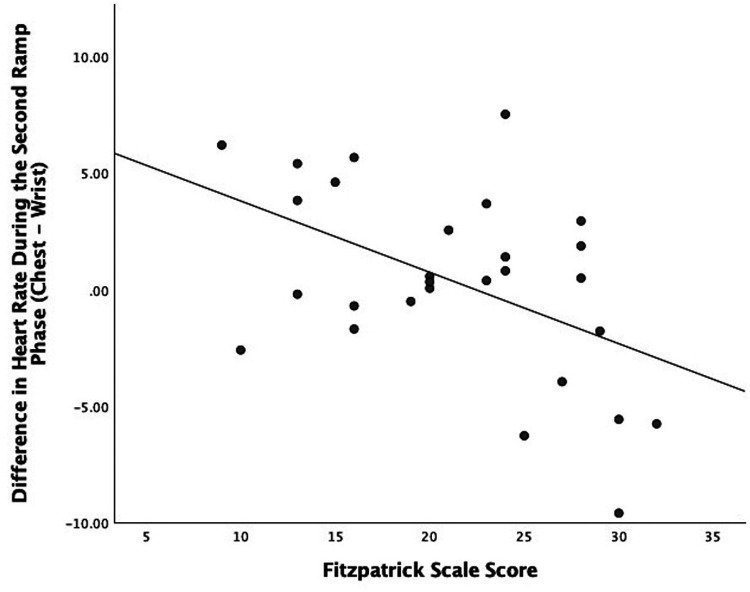
Scatter plot showing the relationship between participant Fitzpatrick scores and the difference between ECG and PPG heart rate values during the second ramp phase.

Regarding Bland–Altman plots (depicted in Figure 1), limits of agreement were >±5 BPM during all tasks. Table 3 depicts the number of cases during each phase with differences that exceeded ±5 BPM. The mean bias was 0.67 BPM during rest (a positive number indicating higher heart rate recorded via ECG relative to PPG), 10.82 BPM during the first ramp phase, 0.20 BPM during the first steady-state exercise phase, 3.49 BPM during the first overall exercise phase, −1.15 BPM during rest, 0.34 BPM during the second ramp phase, −1.11 during the second steady-state exercise phase, and −0.70 during the second exercise bout on the whole. Table 4 displays the Pearson correlation coefficients for the relationships between continuous Fitzpatrick scores and differences in heart rate. There was a significant, moderate negative correlation between Fitzpatrick score and heart rate differences during the second ramp phase, indicating those with darker skin tones demonstrated a relatively higher PPG score during this period. A scatterplot depicting this relationship can be observed in Figure 2. Spearman rho correlations are depicted in Supplementary Table S2, and, notably, interpretation does not meaningfully differ.

**Table 3:** Count of participants exhibiting differences between ECG- and PPG-recorded heart rate exceeding 5 BPM.

Phase	*N* (%)
Start	5 (17)
First ramp	21 (72)
First steady-state exercise	3 (10)
First full exercise bout	8 (28)
Rest	2 (7)
Second ramp	8 (28)
Second steady-state exercise	1 (3)
Second full exercise bout	2 (7)

**Table 4:** Pearson correlations between Fitzpatrick score and differences in heart rate collected via chest and wrist monitor.

Stage	Fitzpatrick score
Start	0.31
First ramp	0.10
First steady-state exercise	0.10
First full exercise bout	0.14
Rest	−0.12
Second ramp	−0.49^a^
Second steady-state exercise	−0.16
Second full exercise bout	−0.28

0–0.2, very weak; 0.2–0.4, weak; 0.4–0.6, moderate ; 0.6–0.8, strong; 0.8+, very strong.

^a^ Correlation is significant at the 0.05 level (two-tailed).

## 5 Discussion

This study aimed to examine the agreement between ECG and wrist-based PPG-measured heart rate, and whether agreement was affected by self-reported skin tone using the Fitzpatrick skin typing scale. Our results generally indicate that ECG- and PPG-measured heart rates did not differ by skin tone, except when individuals were increasing the intensity of activity after an active rest period. Here, those with lighter skin as reported via the Fitzpatrick skin typing scale demonstrated a similar response as to the first ramp phase (i.e., ECG detected higher heart rate) whereas those with darker skin reported relatively higher PPG-recorded heart rate. Importantly, most of these differences were small in magnitude (i.e., 71% had differences <5 BPM, which falls within an acceptable range of accuracy). More generally, we observed a range of bias (1.15–10.82 BPM) between devices, with the greatest bias observed during the initial increase in activity as individuals began their first bout of exercise from complete rest. The tendency for PPG signals to lag behind ECG during changes in heart rate is widely reported and may be attributable to several physiological causes, including a delay between changing heart rate and changes in blood volume at the wrist (14). As others have reported, this suggests utility for wrist PPG-based heart rate measurement for monitoring steady-state activity above, and less so for activities like high-intensity interval training where heart rate might rapidly accelerate and decelerate. This may be especially true for those with darker skin, as we observe a significant correlation between Fitzpatrick score and differences between ECG and PPG only when individuals increased intensity following an active recovery period. Indeed, we observed two outlying individuals who had much greater differences between ECG and PPG ratings during the ramp and/or exercise phases, and both individuals fell into the highest Fitzpatrick category.

In sum, our data, in combination with the wider body of evidence (15, 31–36), give helpful guidance to clinicians and those interested in promoting activity behavior from the perspective of selecting a heart rate monitoring device. Specifically, the use of PPG-based monitoring should be cautioned for those interested in promoting or engaging in behaviors where rapid changes in intensity are expected to be frequent. Moreover, we would note the critical importance of additional validation testing. As raised in recent reports, there remain a number of factors that could interact with skin tone to produce bias, including factors such as the presence of arm hair, ambient temperature and humidity, level of motion, skin thickness, and body mass (31). As researchers gain access to better tools to measure these factors in the field and as consumer wearable devices continue to enter the market, it will be critical to revisit this topic.

### 5.1 Strengths and limitations

There are several important strengths to the present study. First, participants completed study procedures outdoors while walking overground, capturing the potential negative impact of uneven motion and light on the accuracy of wrist data, which may be missed in the laboratory. Additionally, our protocol allowed for the investigation of both steady state and changes in activity intensity. This provides the ability to challenge the validity of these devices over varying conditions and various phases of activity to properly capture every phase of activity that the PPG sensor would record. Of course, there are several important limitations to consider. As described earlier, there are well-documented criticisms of the Fitzpatrick skin typing scale related to how values are interpreted and variability in tone within categories. Given the potential harm produced by biases in widely used health technologies, it is promising that researchers are actively working to create more representative cost-efficient scales, such as the newly developed 10-shade Monk skin tone scale, which became available for use following the completion of data collection for the study presented herein (37), and relatively cost-efficient and portable technologies such as the Delfin Skin Color Catch that researchers have successfully used to observe skin pigmentation (38–40). In combination, these tools may facilitate still further replication work to address several of the research gaps (31). These include investigating interactions between skin tone, arm hair, perspiration, and body mass among other potentially confounding variables. Second, as our research occurred on the campus of a small liberal arts campus, participants were college-aged adults, limiting age diversity in our sample. Extending this work to older adults may cause other discrepancies, as older adults tend to have stiffer arteries, weaker blood flow, and thinner skin (41). Third, our sample was relatively small, which may influence the stability of our findings and the width of our limits of agreement. We acknowledge that a larger, balanced sample size would yield stronger conclusions and also better consider the individual variability in PPG accuracy (25). However, we would note our findings are in line with other recent studies on the topic and are encouraged by the consistent results we observed within our diverse sample (34, 42). Fourth, the discrepancy between PPG and ECG may be subject to motion factors, although this is outside the scope of our study (43). This minor limitation was not assessed in the data processing because all our participants did the same activities, and we are focusing on skin tone. Finally, we did not quantify weather conditions during testing, and evidence suggests that both temperature and humidity may affect the quality of a PPG signal (44). It may be valuable for future research to examine whether there are any interactions between temperature, humidity, and skin pigmentation on PPG accuracy.

## 6 Conclusion

Wearable devices have become a mainstay in clinical trials research and in the consumer sector, and as such, understanding whether and to what extent important characteristics such as skin tone may introduce a bias into heart rate measurement is critical. Herein, we present further support that PPG and ECG-measured heart rates generally exhibit low bias but wide limits of agreement, with differences being exaggerated as activity intensity changes, but generally not varying by the skin tones represented in our sample. These findings are encouraging, supporting the utility of accessible and inexpensive PPG heart rate measurement in health research, especially when one is interested in heart rate at rest or during steady-state activity. Given the rapidity with which widely used wearable technologies advance, it will be critical that researchers routinely replicate research meant to capture any potential bases introduced by the use of these devices.

## Data Availability

The original contributions presented in the study are included in the article/Supplementary Material, further inquiries can be directed to the corresponding author.
