# Supplementary material for: Investigating the accuracy of Garmin PPG sensors on differing skin types based on the Fitzpatrick scale: cross-sectional comparison study
Source: Front Digit Health. 2025 Mar 27;7:1553565. doi: 10.3389/fdgth.2025.1553565 (PMC11983641; doi:10.3389/fdgth.2025.1553565)
Supplement: Supplementary file 1 [file Table1.docx]

**Supplemental Table 1.** Median and Interquartile Range for the difference of heart rate in each task overall and by Fitzpatrick category.

|  | **Fitzpatrick Score** | | |  |
| --- | --- | --- | --- | --- |
|  | 0-13 (N=5) | 14-27 (N=17) | 28-36 (N=7) | **Total (N=29)** |
| **Start** | 2.21 (9.64) | 0.75 (3.66) | 0.11 (2.87) | 0.74 (2.75) |
| **First Ramp** | 17.07 (13.31) | 6.12 (14.77) | 11.95 (18.12) | 10.04 (15.41) |
| **First Steady State Exercise** | -0.33 (3.71) | -0.81 (2.43) | 0.38 (2.75) | -0.33 (2.43) |
| **First Full Exercise Bout** | 5.36 (7.07) | 0.82 (2.95) | 3.77 (6.14) | 2.19 (4.97) |
| **Rest** | -0.67 (1.89) | -1.03 (2.27) | -1.12 (1.69) | -0.93 (2.19) |
| **Second Ramp** | 3.81 (7.19) | 0.47 (4.05)* | -1.79 (7.62) | 0.43 (5.25)* |
| **Second Steady State Exercise** | -0.68 (3.40) | -0.25 (1.37) | -0.53 (2.28) | -0.39 (1.47) |
| **Second Full Exercise Bout** | -0.44 (3.94) | 0.11 (1.45) | 0.08 (4.01) | 0.08 (1.73) |

*Notes: Differences were computed as ECG – PPG such that more positive scores indicate higher ECG-recorded heart rate. Values are in average beats-per-minute. *One observation missing due to lack of clear delineation between ramp and steady state exercise and therefore time was all categorized as exercise.*
