# Supplementary material for: Investigating the accuracy of Garmin PPG sensors on differing skin types based on the Fitzpatrick scale: cross-sectional comparison study
Source: Front Digit Health. 2025 Mar 27;7:1553565. doi: 10.3389/fdgth.2025.1553565 (PMC11983641; doi:10.3389/fdgth.2025.1553565)
Supplement: Supplementary file 2 [file Table2.docx]

**Supplemental Table 2.** Spearman rho correlations between Fitzpatrick score and differences in heart rate collected via chest and wrist monitor.

| **Stage** | **Fitzpatrick Score** |
| --- | --- |
| Start | 0.08 |
| First Ramp | 0.03 |
| First Steady State Exercise | 0.13 |
| First Full Exercise Bout | -0.02 |
| Rest | -0.18 |
| Second Ramp | -0.42* |
| Second Steady State Exercise | -0.13 |
| Second Full Exercise Bout | -0.24 |

*. Correlation is significant at the 0.05 level (2-tailed).

0 - .2 very weak

.2-.4 weak

.4-.6 mod

.6-.8 strong

.8+ very strong
